# *Stenotrophomonas maltophilia* Necrotizing Soft Tissue Infection in an Immunocompromised Patient

**DOI:** 10.1155/2018/1475730

**Published:** 2018-04-01

**Authors:** Oleg Stens, Gabriel Wardi, Matthew Kinney, Stephanie Shin, Demosthenes Papamatheakis

**Affiliations:** ^1^Department of Internal Medicine, Harbor-UCLA Medical Center, Torrance, CA, USA; ^2^Department of Emergency Medicine, University of California, San Diego, San Diego, CA, USA; ^3^Division of Pulmonary, Critical Care, and Sleep Medicine, University of California, San Diego, La Jolla, CA, USA; ^4^Department of Orthopedic Surgery, University of California, San Diego, San Diego, CA, USA; ^5^Pulmonary and Critical Care Medicine, Sharp Memorial Hospital, San Diego, CA, USA

## Abstract

**Introduction:**

To report on the first recorded case of necrotizing soft tissue infection (NSTI) in an immunocompromised individual caused by* Stenotrophomonas maltophilia* in the Western Hemisphere and highlight the challenges that medical providers face in promptly diagnosing and treating NSTI in this highly vulnerable patient population.

**Case Presentation:**

We report a case of NSTI caused by* S. maltophilia* in a neutropenic patient admitted for treatment of acute lymphoblastic leukemia. The patient presented with laboratory and clinical findings atypical for a NSTI that may have confounded its diagnosis and delayed surgical intervention. Despite aggressive medical care and surgical debridement, the patient unfortunately passed away due to overwhelming septic shock.

**Conclusions:**

Providers should consider atypical organisms as causative in NSTI in immunocompromised patients and recognize that these patients may present without classic clinical and laboratory findings.

## 1. Background

The term necrotizing soft tissue infection (NSTI) describes an infection caused by bacteria or fungi that spreads rapidly along tissue planes, causing vascular occlusion and necrosis, and carries high mortality even when promptly identified. These infections are rare, with approximately 1000 cases per year reported in the United States, although it is generally felt that the number of cases is underreported and is on the rise [1, 2]. Scoring systems, such as the laboratory risk indicator for necrotizing soft tissue infection (LRINEC), have been developed to help providers differentiate between NSTI and severe cellulitis [3]. However, the interpretation of this score in immunocompromised patients and particularly those with hematologic malignancies is challenging as common laboratory and physical exam findings may be absent. Furthermore, these patients are at high risk for atypical organisms that may require alternative antibiotic regimens in addition to prompt surgical intervention. To illustrate this, we report a case of an individual with acute lymphoblastic leukemia who developed an NSTI from* Stenotrophomonas maltophilia*, the first case of this being reported in the Western Hemisphere, which illustrates the unique challenges in the identification and management in this patient population.

## 2. Case Presentation

A 39-year-old otherwise healthy male with newly diagnosed mixed-lineage acute monocytic and B-cell lymphoblastic leukemia was transferred to our institution for initiation of chemotherapy. Prior to transfer, he had received intrathecal methotrexate and oral dexamethasone, as well as vancomycin and cefepime for neutropenic fever and positive blood cultures growing gram-positive cocci. At our institution, the patient was started on GRAALL (Group for Research on Adult Acute Lymphoblastic Leukemia) 2003 chemotherapy. He received cefepime and vancomycin for persistent neutropenic fevers. Eventually, cefepime was switched to meropenem due to development of multifocal pneumonia during his hospital course. Shortly thereafter, liposomal amphotericin was initiated for presumed invasive pulmonary aspergillosis after a bronchoalveolar lavage (BAL) from a diagnostic bronchoscopy revealed a positive galactomannan antigen; however no organisms were seen on gram stain or identified on culture. He was treated with vancomycin for 8 days and meropenem for 17 days, after which he defervesced. Nevertheless, fevers recurred on hospital day 20 and shortly after; blood cultures returned with gram-negative rods at which point vancomycin and cefepime were reinitiated.

**Figure 1:**
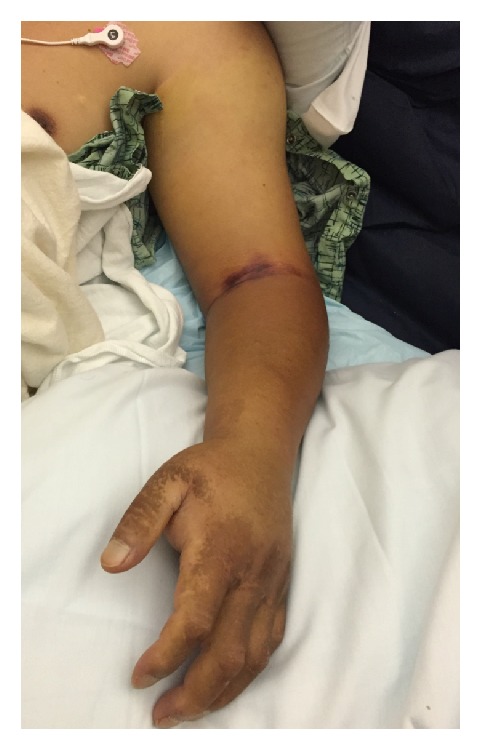
Appearance of the patient's arm prior to surgery.

Three days later (hospital day 23), the patient reported pain and developed swelling and erythema of his left forearm. On physical examination, the left forearm was swollen, tense, and erythematous, but there were no areas of fluctuance, subcutaneous crepitance, or bullae noted. Duplex ultrasound of the upper extremities showed no evidence of deep vein thrombosis. However, given the acute onset of pain and swelling, orthopedic surgery was consulted for possible necrotizing infection and compartment syndrome. Laboratory testing disclosed a c-reactive protein (CRP) of 12.90 mg/dL, a white blood cell count of less than 0.1 × 10^9^/L, a sodium of 142 mmol/L, a glucose of 110 mg/dL, a hemoglobin of 7.2 g/dL, and a creatinine of 0.96 mg/dL, yielding a LRINEC score of 2 [3]. It was felt, partly due to this low score, that the patient had a severe cellulitis without necrotizing infection or compartment syndrome. Moreover, based on the patient's clinical picture and the available data at the time, invasive testing for compartment syndrome was deemed unnecessary by the orthopedics service. No additional imaging was ordered at this point. Within 10 hours, the swelling and pain had progressed to his entire left upper extremity (Figure 1) and the patient developed hypotension. He was then transferred to the intensive care unit for refractory septic shock and severe metabolic derangement. At this time, laboratory studies were notable for a CRP of 18.80 mg/dL, a white blood cell count of 0.2 × 10^9^/L, a sodium of 142 mmol/L, a glucose of 116 mg/dL, a hemoglobin of 5.9 g/dL, and a creatinine of 1.06 mg/dL yielding a LRINEC score of 6. The patient continued to clinically decline rapidly despite broadening of his antibiotic regimen (vancomycin, meropenem, gentamicin, and amphotericin B) and was taken to the operating room by the orthopedic surgery service due to concern of a rapidly progressing NSTI.

**Figure 2:**
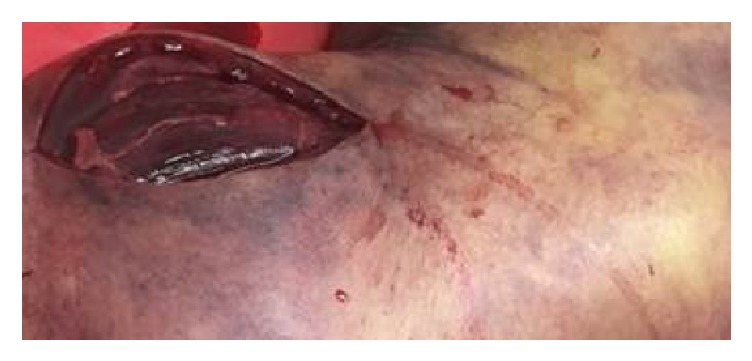
Intraoperative photograph showing nonviable tissue from incision made over the biceps muscle.

In the operating room, the entire left upper extremity was prepped, and an initial incision was made over the dorsum of the hand. Exploration of the surrounding tissue revealed necrotic fat, hemorrhagic changes, and gross edema. The expressed fluid was tan but did not have the classic “dishwater” appearance of necrotizing fasciitis, nor was it malodorous. The fascia was thickened and opaque. A counterincision was then made proximally over the biceps muscle in the proximal brachium (Figure 2). This region displayed the same tan drainage with associated fat necrosis, and thrombosed vessels were also appreciated in the subcutaneous tissue. Regions of the thickened, diseased-appearing fascia were excised, revealing that the underlying musculature appeared necrotic, noncontractile, and nonviable.

At this point during the procedure, the anesthesiologist noted that multiple vasopressors were being used to maintain the patient's blood pressure, and there was concern that he was clinically deteriorating. Given the extensive involvement of, essentially, the entire limb and the concern that the bacterial load could not be adequately controlled in this hemodynamically unstable patient, the decision was made to proceed with an emergent glenohumeral amputation. The deltoid muscle remained healthy-appearing and contractile, and thus a lateral-based flap composed of the deltoid and overlying skin and soft tissue was planned. Once the amputation was performed, the wound was copiously irrigated using several liters of normal saline to minimize the remaining bacterial load. Hemostasis was subsequently achieved, a drain was placed, and the flap closure was completed. The patient's hemodynamic status improved during closure, and he was transferred back to the intensive care unit for continued care.

The previously drawn blood culture returned on postoperative day (POD) 0, growing* Stenotrophomonas maltophilia* in both aerobic and anaerobic cultures, at which point trimethoprim-sulfamethoxazole was initiated. Pathology of the surgical specimen confirmed the diagnosis of a NSTI, and multiple tissue cultures grew only* S. maltophilia*. Despite appropriate antibiotic therapy and surgical intervention, the patient remained persistently bacteremic and in refractory shock despite aggressive medical therapy. On POD1, new necrotic skin areas were noted at the surgical site, and cardiothoracic surgery was consulted to evaluate further surgical intervention. The team decided against this, given the patient's very poor prognosis. After discussion with the family, the patient was transitioned to comfort care and passed away shortly thereafter.

## 3. Discussion

In this article, we present a case of a monomicrobial NSTI caused by* Stenotrophomonas maltophilia* in a neutropenic patient with hematologic malignancy. To our knowledge, this is the first reported case of a NSTI due to* S. maltophilia* in the Western Hemisphere. Sakhnini and colleagues from Israel reported two young women on the same hospital ward dying from fulminant* S. maltophilia* soft tissue infection of the extremities [4]. The key similarities between all three cases include neutropenia (the two women in Israel were neutropenic due to acute myeloid leukemia and aplastic anemia, resp.) and empiric treatment with a carbapenem-class antibiotic prior to the onset of soft tissue infection. We suggest that correctly diagnosing NSTI in immunocompromised patients is particularly challenging, since the causative organisms and the clinical features may differ significantly compared to immunocompetent patients.

NSTIs are classified based on the microbiology of the infection. Type I NSTIs, by far the most common, are polymicrobial, with both aerobic and anaerobic organisms typically present. Type II NSTIs are monomicrobial infections typically associated with gram-positive pathogens like *β*-hemolytic* streptococci* or* S. aureus.* Type III NSTIs are the least common type and are typified by* Clostridium* species, which cause rapidly progressive myonecrosis. Finally some necrotizing infections do not fall within the normal classification system, such as the waterborne agent-related NSTIs (*Aeromonas hydrophila* and* Vibrio vulnificus*) and invasive rhinocerebral fungal infections [5–7]. Prompt diagnosis and surgical intervention are essential to patient survival. Even with early diagnosis, surgical debridement, and aggressive supportive care, mortality remains high, estimated to be over 20% [7]. Unfortunately, there is no single imaging or laboratory study that is both sensitive and specific for NSTIs. The LRINEC score was introduced in 2004 as a method to help providers differentiate between severe cellulitis and NSTIs [3]. Although the original study showed promising positive and negative predictive values for this score [3], follow-up validation studies showed lower sensitivities, challenging the scores ability to distinguish NSTI [8, 9].

In addition to the above, this score may be even more misleading in myelosuppressed patients, since leukocytosis would be absent and anemia could be attributed to impaired erythropoiesis. Furthermore, the characteristic intraoperative finding of a NSTI, “dishwater” discharge, maybe absent in these patients, likely due to profound neutropenia. In our case, the initial LRINEC score of 2 was misleading and provided false reassurance. Moreover, the delay in speciation of the gram-negative rod noted on blood cultures also led to inappropriate antibiotic coverage, despite broad-spectrum antibiotics being provided.

Unfortunately, the diagnosis of NSTIs in immunocompromised populations has been poorly studied. A single-center retrospective study conducted at Brigham & Women's Hospital in Boston found that immunocompromised patients with NSTI were less likely to have significant leukocytosis or purulent drainage at the site of infection, compared to immunocompetent NSTI cases. The immunosuppressed patients NSTI were also more likely to experience delays in diagnosis and initial surgical debridement, which were associated with worse outcomes [10].

*S. maltophilia* is a waterborne, gram-negative, obligate aerobe bacterium that has been isolated from a variety of sources in the hospital settings. It is an opportunistic healthcare-associated infection closely related to* Pseudomonas* and has high morbidity and mortality in certain patient populations, particularly the immunosuppressed ones. The name* Stenotrophomonas* comes from the Greek language and means “a unit feeding on few substrates” whereas* maltophilia* means “a friend of malt” [11].* S. maltophilia* has been known to cause a wide range of infections, including pneumonia, bacteremia, endocarditis, and meningitis [12]. Skin and soft tissue infections by* S. maltophilia* including metastatic and primary cellulitis and ecthyma gangrenosum have also been reported, predominantly in patients receiving chemotherapy for hematologic malignancies [13–15]. In our patient, we would favor a primary cellulitis as the source of the infection given that pain and swelling of the left upper extremity preceded clinical deterioration, and there were no other localizing symptoms. Respiratory cultures were negative for the organism. However, we cannot rule out metastatic soft tissue infection from endocarditis as echocardiography was never obtained. Our case report highlights a NSTI due to a microorganism uncommonly associated with such an infection and raises concerns for the breadth of pathogens that can cause such invasive infections on immunosuppressed patients.

Treating infections caused by* S. maltophilia* is challenging because of the organism's inherent resistance to a broad range of antibiotics, including almost all beta-lactams, carbapenems, cephalosporins, and aminoglycosides. Resistance to carbapenems is particularly problematic in the intensive care unit as this class of antibiotic is frequently used when patients fail to respond to other antibiotics that cover gram-negative infections. Trimethoprim-sulfamethoxazole is presently the antibiotic of choice, although ticarcillin-clavulanic acid, minocycline, tigecycline, and certain fluoroquinolones may also be effective [16]. Because of its antibiotic resistance,* S. maltophilia* is not covered by most empiric antibiotic regimens and requires a high level of suspicion to consider this diagnosis and provide proper treatment. More recently,* S. maltophilia* infection has become increasingly common in cancer patients and has been possibly associated with carbapenem antibiotic use, to which it is resistant [17–21].

## 4. Conclusion

NSTIs in immunocompromised patients present a unique challenge. These patients may not present with the laboratory abnormalities and physical exam findings typically associated with NSTI, leading to a delay in diagnosis and possibly worse outcomes. Moreover, causative organisms may be atypical for NSTI, compared to immunocompetent patients, which may impede appropriate antibiotic therapy. Of these,* S. maltophilia* is becoming increasingly important, based on higher incidence in certain populations. Overreliance on scoring tools such as LRINEC should be avoided, particularly in special patient populations such as the immunocompromised ones and those with hematological malignancies as the patient's underlying disease process may affect results. Potential future research can include targeted prophylactic antibiotic use in certain patient populations that are at higher risk for atypical NSTI. This could include patients with a hematological malignancy or patients who develop new infectious symptoms after receiving carbapenems or other broad-spectrum antibiotics.

## Abbreviations

NSTI: Necrotizing soft tissue infection
GRAALL: Group for Research on Adult Acute Lymphoblastic Leukemia
CRP: c-reactive protein
LRINEC: Laboratory risk indicator for necrotizing soft tissue infection
POD: Postoperative day.
